# Research on MOOC Teaching Mode in Higher Education Based on Deep Learning

**DOI:** 10.1155/2022/8031602

**Published:** 2022-01-29

**Authors:** Yuan Tian, Yingjie Sun, Lijing Zhang, Wanqiang Qi

**Affiliations:** ^1^Aviation University of Air Force, Changchun, Jilin 130022, China; ^2^School of Automotive Engineering, Jilin Teachers Institute of Engineering and Technology, Changchun, Jilin 130022, China

## Abstract

With the rapid development of computer technology and network technology and the widespread popularity of electronic equipment, communication among people is more dependent on the Internet. The Internet has brought great convenience to people's lives and work, and the Internet data is constantly being recorded. People's data information and behavior information, which provides the basis for data mining and recommendation systems, mining users' information and behaviors, and providing “user portraits” for each user, can provide better services to users and it is also an important part of the recommendation system. In one step, this article takes MOOC education resources as the research goal. In order to improve the effective management of MOOC platform resources based on traditional methods, this article combines relevant data sets and recommendation techniques to initially build a learning platform, implements a deep neural network algorithm, and recommends related services. The request and response data were explained, and through the online learning data set, based on the learner's historical learning records, the learning resources were simulated and recommended to the learners. The resource customization module was elaborated. Through the results of resource recommendation, a personalized learning resource recommendation platform was initially realized, which more intuitively demonstrated the recommendation effect and better realized the teaching management of the MOOC platform.

## 1. Introduction

MOOC stands for “Massive Open Online Course,” which is an open-access and large-scale participation online course. This brand-new course model was opened by Professor David Wiley of Utah State University in the United States and Professor Alec Couros of the University of Regina in Canada. The two Internet open courses developed and evolved. In recent years, “Internet+” education has been surging. With the advantages of breaking time and space constraints and integrating and optimizing learning resources, MOOC has gradually gained widespread attention and developed rapidly in China. MOOC has changed the traditional classroom structure where teachers mainly teach and changed the single mode of acquiring new knowledge through books, making the identities of teachers and students fundamentally change in the teaching process. The most popular teaching reform in today's education field is the rise of large-scale open online course MOOC platforms. We have investigated the types of courses offered on the existing MOOC platforms in China and found common problems. The course resources of the major platforms are not balanced. They mainly offer engineering theoretical courses but lack experimental courses corresponding to engineering theoretical courses. According to the statistics of the “Chinese University Online Open Course Forum,” there are currently more than 1,300 online courses on the “China University MOOC” platform, the largest open online course platform in China, and more than 20 million courses have been selected. However, according to the syllabus of engineering disciplines, there are more than 360 courses that need to be opened, but only 11 results of online experimental courses were actually searched, which only accounted for 3.05% of the total number of experimental courses specified in the syllabus. The proportion of experimental courses offered by various platforms is seriously unbalanced [1–5].

With the rapid development of the MOOC platform, more and more college students and social learners choose courses, and college teaching also attaches great importance to the design of online courses. Especially for engineering courses, the courses of some colleges and universities have supported teaching links such as credit certification, homework release, course performance evaluation, and academic certificate issuance. The final subject scores are evaluated based on the online teaching process, which completely replaces the traditional classroom teaching model. Judging from this development trend, it also fully illustrates the importance of course content design under the online platform. However, online learning led by MOOC still faces many doubts. Among them, the most criticized is that there are not many learners who insist on completing the courses, and the dropout rate remains high. Online learning does have a huge impact on traditional teaching with its unique advantages, but we also need to note that the nature of online learning determines that teachers and students are separated in time and space, and teachers cannot observe and learn as intuitively as in traditional classrooms. As a result, the learning behavior of the learner cannot detect and feed back problems in time and give early warning. Therefore, based on the theory of social cognition, this research focuses on the learning environment and learning psychology of the two MOOC learning context elements and constructs a MOOC learning behavior model oriented by the cognitive participation of learning behavior. The relationship between the effects is analyzed in depth, and the teaching rules and pedagogical principles are explained. Finally, based on the model, the deep neural factorization machine is used to predict the learning effects of MOOC learners.

## 2. Related Theoretical Methods

### 2.1. Online Learning Behavior

Compared with the learning behaviors generated in traditional classrooms, MOOC online learning behaviors have their own outstanding characteristics. They are not restricted by space, learning resources are richer, and learning is more intelligent and efficient. During the interaction between learners and the Internet, the behaviors generated are easy to collect, mine, and analyze, which can provide data and theoretical basis for the improvement of the level of online education in the future.

The effective learning behaviors of MOOC learners mainly include the following:

1. *Watch the Course Video.* Watching the course video is the main means for learners to learn in MOOC. Through the learning of video resources, learners can follow the teacher to intuitively learn the content of the course.
2. *Submit Homework and Test.* In the learning platform, as the course progresses, teachers will assign some homework for learners to answer. The homework can not only check the learner's learning effect in time, but also broaden the learner's horizons, so that the learner can summarize and reflect. With the increase in learning content, teachers hope to understand learners' learning effects through tests and examinations and at the same time, through tests and examinations, to give learners the opportunity to check for deficiencies.
3. *Participate in Exchanges in the Discussion Forum.* After the learners have finished learning the video of the course, they may still have doubts about some of the problems of the course. At this time, they can go to the discussion area corresponding to the course to search for the problem and see if any other learners have encountered the same problem. You can also post by yourself, seek help from other learners, or answer questions raised by other learners in the forum to help everyone make progress together [6–10].
4. *Other Learning Behaviors.* When the learner is studying the MOOC course, in addition to the learning of video resources, the teacher will also provide learners with other forms of learning resources, such as courseware, bibliography, sample codes, etc., which can be used as a supplement to video learning, which is very important learning behavior.

In order to analyze and predict learners more accurately, 11 learning behavior characteristics that can represent their learning behavior characteristics are selected from the behavior logs generated by the learners in the MOOC learning process. These 11 learning characteristics are selected from the number of activities. Look at the video situation, homework situation, forum discussion situation, and other dimensions to describe the learner's learning behavior. The names and descriptions of the 11 learning behavior characteristics are shown in Table 1.

### 2.2. Text Classification on MOOC Platform

**Figure 1:**
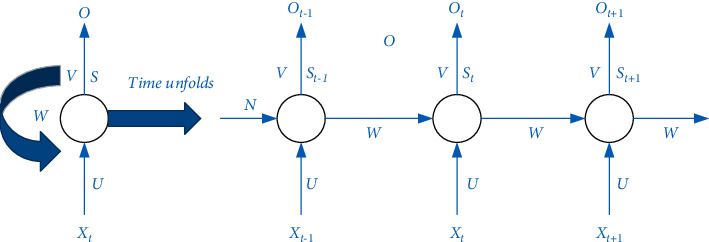
Standard structure diagram of cyclic neural network.

This article mainly uses LSTM in deep learning to classify texts in MOOC platform teaching management and innovates on the basis of RNN neural network model. The biggest difference from other models is that the biggest feature of recurrent neural network is that it propagates in the forward direction. In the process, the data obtained by the current neuron is affected not only by the current input data but also by the output data of the previous node. Based on this feature, the cyclic neural network can better process data with time series attributes. The standard RNN network structure is shown in Figure 1, where *X* represents the input, *O* represents the network output, U, V, and W, respectively, represent the weights between the layers, and S represents the output of the hidden layer in the network [11–13].

**Figure 2:**
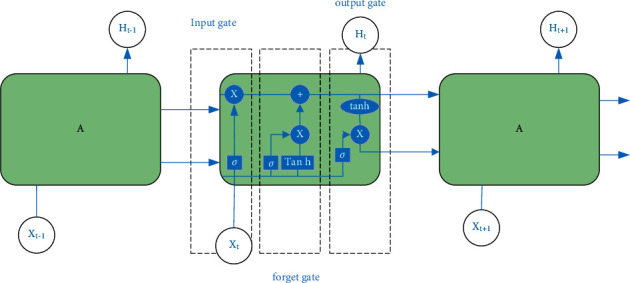
LSTM network structure diagram.

Numerous experiments have shown that RNN has certain shortcomings for dealing with long-time sequence problems. The longer the time series is, the weaker the memory capacity of the RNN will become, which will cause the network to be unable to save the previous output information when subsequent neurons are trained. In order to solve such problems, the researchers put forward an LSTM model that can record long-term information through experiments and demonstrations. Early methods to solve these problems were either tailored to specific problems or did not extend to long-term dependencies. Only LSTM is both versatile and effective in capturing long-term time dependencies. LSTM implements the screening of information through the “gate” control structure. The “gate” is represented as a fully connected layer in the network. The standard LSTM core unit consists of an input gate, an output gate, and a forget gate. With 3 kinds of gate control composition, each gate has a different function, the input gate is mainly responsible for determining how much valid input information is retained, the output gate determines how many valid unit states are output at the current moment, and the forget gate determines whether to retain the previous one. The specific network structure of the status output information is shown in Figure 2 [14].

### 2.3. Evaluation Index of the Algorithm

TOP-N recommendation is to recommend the Top-N items that users of the MOOC platform may like. Generally, accuracy and recall rates are commonly used to measure the effect of Top-N recommendation. The specific definition and expression are as follows:

*R*(*u*) represents the recommendation list obtained by the target user on the training set, and *T*(*u*) represents the recommendation list obtained by the user on the test set. According to the above information, the recall rate is expressed as

(1) $$\begin{array}{c} \text{recall}=\frac{\sum_{u\in U}\left|R\left(u\right)\cap^{}T\left(u\right)\right|}{\sum_{u\in U}\left|T\left(u\right)\right|}. \end{array}$$

The accuracy of the recommended results is expressed as

(2) $$\begin{array}{c} \text{precision}=\frac{\sum_{u\in U}\left|R\left(u\right)\cap^{}T\left(u\right)\right|}{\sum_{u\in U}\left|R\left(u\right)\right|}. \end{array}$$

Novelty is defined as the reciprocal of popularity. If a product is very popular, it appears more frequently, which is not a novel recommendation result for users. It is expressed as formula (3), where *K*_*c*_ represents the popularity of the product.

(3) $$\begin{array}{c} \text{novelty}=\frac{1}{\log_{2}\left(K_{C}+1\right)+1}. \end{array}$$

In the Top-N recommendation, the *F*1 comprehensive evaluation index is also the ratio of the precision rate and the recall rate, expressed as

(4) $$\begin{array}{c} F1=\frac{2^{*}{\text{precision}}^{*}\text{recall}}{\text{precision}+\text{recall}}. \end{array}$$

## 3. Recommendation Algorithm Model Construction Based on MOOC Platform Learner Model

### 3.1. MOOC Platform Learner Model Construction

Constructing the learner model mainly includes two parts: the collection of learner behavior characteristics and the construction of the learner model.

1. Collection of learner behavior characteristics: before establishing a learner model, learner behavior characteristics need to be extracted. The model mainly includes learner's personal information characteristics and learner's learning characteristics, as shown in Table 2.
2. MOOC learner model construction: the personal characteristics of learners mainly include registration information, learning ability, and historical learning content. The learning characteristics of learners include learning courses, learning time, learning experience, learning scores, learning progress, number of homework submissions, course scores, and learning ability [15–18].

**Figure 3:**
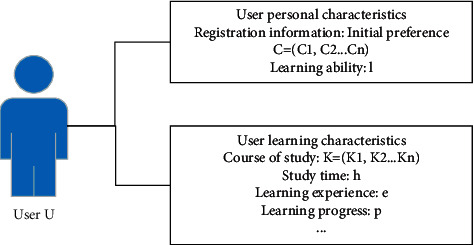
Diagram of the learner model.

Taking Figure 3 as an example, combined with the above analysis, define *U* as a learner, the preference obtained by analyzing the registration information is *C* = {*C*_1_, *C*_2_,…, *C*_*n*_}, the learning course is *K* = {*K*_1_, *K*_2_,…, *K*_*n*_}, and the learning ability is *l*, the learning time is *h*, the learning experience is *e*, the learning score is *s*, and the learning progress is *p*.

### 3.2. Overall Description of Recommendation Algorithm Based on MOOC Platform Learner Model

**Figure 4:**
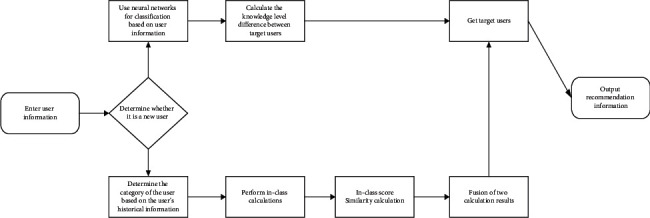
The overall flowchart of the learner model.

The overall structure of the learner model of the MOOC platform is shown in Figure 4. The recommendation algorithm based on the learner model mainly solves two problems. The algorithm is described as follows:

1. At the new user level, the cold start is alleviated by using the neural network to automatically extract user interests, and the user's favorite course category is obtained through the user's interest, and then the learning ability similarity between users is calculated within the category to obtain similar users for recommendation;
2. At the old user level, the user category is determined through user history information, and the learner's learning behavior characteristics and learning ability similarity are introduced for intraclass calculation to obtain the model similarity score. On this basis, the intraclass user score similarity is calculated, and finally, fusion calculation model similarity score and scoring similarity score are calculated to get the final similar users to recommend [19, 20].

### 3.3. Using Neural Networks to Alleviate Cold Start

The LSTM_Max-pooling text classification method is used to analyze the registration information of new users to obtain the interest categories of the new users and provide a basis for the next resource recommendation.

#### 3.3.1. Implementation of MOOC Platform Text Classification Based on LSTM_Max-Pooling

**Figure 5:**
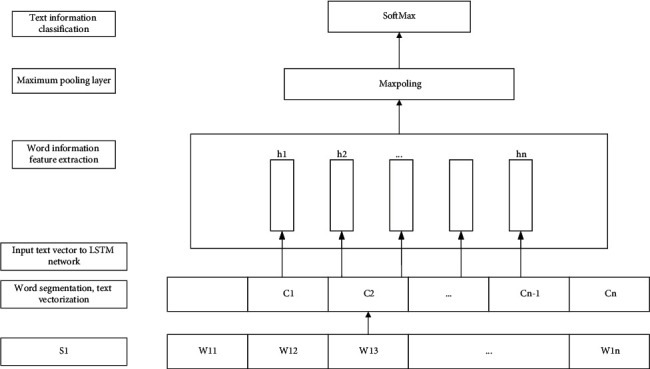
LSTM_Max-pooling classification model diagram.

This article uses the LSTM_Max-pooling classification model shown in Figure 5. The network model unit consists of 5 layers. The first layer is the embedding layer for word segmentation and vectorization operations; the second layer is spatial_dropout1d to improve the independence between features; the third layer is an LSTM layer, which contains 100 neurons. The input and output activation functions use tanh, and the “gate” activation function uses sigmoid; the fourth layer uses Max-pooling to obtain the most salient features and make the model suitable for short text classification; the fifth layer is a fully connected layer using Softmax for classification. In order to prevent the occurrence of overfitting, dropout is used in the training process and the generalization ability of the model is improved. The model workflow is as follows:

  Step 1: complete sentence segmentation and vectorization
  Step 2: take the word vector as input, and output the vector *h*_1_, *h*_2_,…, *h*_*n*_ of the hidden LSTM neural unit at *n* times through the LSTM layer processing
  Step 3: input the vector output from the upper layer for maximum pooling operation to the feature vector *h*
  Step 4: the upper layer feature vector *h* is processed by the Softmax layer to complete the text information classification [21, 22]

#### 3.3.2. Calculating the Similarity of the Learning Ability of Users on the MOOC Platform

**Definition 1 .**

*C* represents the category of course resources, *C* ∈ {*C*_1_, *C*_2_,…, *C*_*n*_}, which maps the user's interest category according to the course category; *L* represents the learning ability level of the learner; *L* ∈ {1, 2,…, 3}1, 2, 3 correspond to elementary, intermediate, and advanced levels, respectively. Newly registered users are beginner users, and registered users are evaluated according to the difficulty classification in their history learning courses; *U* means all users, *U*_*Cj*_ means the *j*-th user in category *C*, and *U*_*Ct*_ means target users. The calculation of the similarity within the new user class is reflected by the similarity of the learning level, and the similarity of the calculated learning level can be expressed using a standard Gaussian distribution, as shown in the following formula:

(5) $$\begin{array}{c} \text{sim}{\left(U_{ct},U_{cj}\right)}_{\text{level}}=\frac{1}{2\pi }e^{-\left(\left(U_{ctL}-U_{cjL}\right)/2\right)}. \end{array}$$

### 3.4. Improving the Similarity Calculation Using the Learner Characteristics of the MOOC Platform

#### 3.4.1. Extracting Interest Tags of Old Users of the MOOC Platform

According to the historical information of the user, the category of all the learning resources browsed by the user is obtained, and the historical learning information of the user is calculated by the statistical method to obtain the user's interest tag. The calculation method is expressed as

(6) $$\begin{array}{c} \text{hoby}\left(U_{t}\right)=\frac{U_{\text{th}C_{i}}}{U_{\text{th}}}i\in \left\{1、2\ldots n\right\}. \end{array}$$

In formula (6), *U*_th*C*_*i*__ represents the number of times the user *U*_*t*_ has learned the *i*-th type of course in the historical information, and *U*_*t*_ represents the total number of times the user has learned.

#### 3.4.2. In-Class Calculation of Learner Model on MOOC Platform

Calculate the user interest category based on the user interest tags extracted in the previous step. The specific process is as follows:

*(1) Using the Learning Features of Old Users inTable* 2 *to Calculate the Similarity of Learning Features*. *U* represents all users, *U*_*ci, t*_ represents the target user, and *U*_*ci, j*_ represents other users in the same category, where *t* ∈ *U*, *j* belongs to *U*. Use two users to calculate the behavior characteristics of the same course, *U*_*ci, t*_ ⟶ *K* represents the behavior characteristics of user *U*_*ci, t*_ on course *K*, *U*_*ci, j*_ ⟶ K represents the behavior characteristics of user *U*_*ci, j*_ on course *K*, *K* is for the courses that two users have studied together, and the behavior of the users on the courses is represented by a row vector, which is expressed as

(7) $$\begin{array}{c} U_{ci,t}⟶K\left[K_{h},K_{e},K_{s},K_{p},K_{z}\right],\\ U_{ci,j}⟶K\left[K_{h},K_{e},K_{s},K_{p},K_{z}\right]. \end{array}$$

In formula (9), *K*_*h*,_*K*_*e*_, *K*_*s*,_*K*_*p*,_ and *K*_*z*_, respectively, represent the proportion of the learner's time to study the course *K* in the total time, the proportion of the experience of obtaining the course *K* in the total experience, the proportion of the score of the study course *K* in the total score, the proportion of the progress of learning course *K* to the total progress, and the proportion of the number of homework submissions to the total number of times. In order to calculate the similarity of courses, use the following formula:

(8) $$\begin{array}{c} \text{sim}{\left(U_{ci，t},U_{ci，j}\right)}_{\text{feature}}=\frac{\sum_{i=1}^{n}U_{ci，j}⟶K_{l}-U_{ci，t}⟶K_{l}}{n}. \end{array}$$

In formula (8), *l* represents the course *l* ∈ {1, 2,…, *n*} that two users have learned together, and *n* represents the total number of courses that two users have studied together. Combine formulas (5) and (8) to calculate learner model similarity, which is expressed as

(9) $$\begin{array}{c} \text{sim}{\left(U_{t},U_{j}\right)}_{\text{model}}=\text{sim}{\left(U_{ci，t},U_{ci，j}\right)}_{\text{feature}}+\text{sim}{\left(U_{ct},U_{cj}\right)}_{\text{level}}. \end{array}$$

*(2) Calculation of Similarity of User Resource Scores*. Calculate the category of the target user's perceptual interest in the target user's historical information, find other users with the same interest as the target user, and use the user ID as the row index and the course ID as the column index to construct a “user-rating” table. Each row in the scoring table represents user's rating information for all courses, each column represents the user's rating obtained by a course, the data in the table indicates the specific rating, and the “user-rating” table is shown in Table 3.

In Table 3, the user set is *U* ∈ {*U*_1_, *U*_2_,…, *U*_*n*_}, and the course resource set *I* ∈ {*I*_1_, *I*_2_,…, *I*_*n*_} is used to calculate the similarity of scores between users. sim(*U*_*t*_, *U*_*i*_)_score_ represents users *U*_*t*_ and *U*_*i*_. The similarity of *i* represents the courses that are jointly graded, and ¯ $\bar{R_{U_{j}}}$ and $\bar{R_{U_{i}}}$, respectively, represent the mean value of user *U*_*t*_ and user *U*_*i*_ in the common scoring item.

(10) $$\begin{array}{c} \text{sim}\left(U_{t},U_{i}\right){}_{\left.\text{score}\right)}=\frac{\sum_{i\in U_{i}，U_{j}}^{n}\left(R_{t，i}-\bar{R_{t}}\right)\left(R_{j，i}-\bar{R_{t}}\right)}{\sqrt{\sum_{i\in U_{i}}^{n}{\left(R_{t，i}-\bar{R_{t}}\right)}^{2}}\sqrt{\sum_{i\in U_{j}}^{n}{\left(R_{j，i}-\bar{R_{i}}\right)}^{2}}}. \end{array}$$

### 3.5. Using Recommendation Algorithm Based on MOOC Platform Learner Model for Recommendation Learning

Through the above description and derivation, the learner model similarity and the score similarity are finally calculated by fusion and addition, which is specifically expressed as

(11) $$\begin{array}{c} \text{sim}{\left(U_{t},U_{i}\right)}_{f}=\text{sim}{\left(U_{t},U_{i}\right)}_{\text{model}}+\text{sim}{\left(U_{t},U_{i}\right)}_{\text{score}}. \end{array}$$

According to the calculation result of formula (11), sort from high to low, and take the first *N* as the neighbor set. Draw the direct neighbor user set, predict the scores of the courses for which the target user does not produce scores among the final neighbor users, select the Top-N courses with the highest predicted scores for recommendation, and the calculation is expressed as

(12) $$\begin{array}{c} p_{U_{t}，i}=\bar{R_{U_{t}}}+\frac{\sum_{U_{j}\in s\left(U_{i}\right)}^{n}\left(R_{U_{i}，i}-\bar{R_{U_{j}}}\right)\cdot \text{sim}\left(U_{t},U_{j}\right)}{\sum_{U_{j}\in s\left(U_{i}\right)}^{n}\left\lceil \text{sim}\left(U_{t},U_{j}\right)\right\rceil }. \end{array}$$

In formula (12), *P*_*ut, i*_ represents the predicted score of user *U*_*t*_ on the unrated item *i*, $\bar{R_{U_{j}}}$ represents the average score of user *U*_*t*_, $\bar{R_{U_{j}}}$ represents the average score of user *U*_*j*_, *R*_*Uj, i*_ represents *U*_*j*_'s score on item *i*, and *s*(*U*_*t*_) represents the neighbor set of user *U*_*t*_.

## 4. Experiment and Result Analysis

### 4.1. Algorithm Evaluation Index

For details of the algorithm evaluation index, refer to the evaluation method and index of the algorithm in 2.3. This experiment uses the accuracy rate, recall rate, and *F*1 comprehensive evaluation index of the Top-N recommended method as the measurement standards.

### 4.2. Designing a Comparative Experiment

The experiment mainly includes two comparative experiments, (1) using different natural language processing methods to classify new users; (2) using traditional recommendation methods on the data set of this article to conduct comparison experiments with the recommendation algorithm proposed in this chapter.

**Experiment 1 .**

The influence of different natural language processing algorithms on the accuracy of user interest extraction. The experiment uses the DataALL data set, which includes 50,000 users and 861 courses. The data set includes the explicit behavior information and implicit behavior of users on the courses. Information is used to verify the accuracy of the algorithm mentioned in Chapter 3 and the accuracy of the three network models of CNN, GRU, and LSTM under the same data size.

By preprocessing the data set, null values and invalid data are removed. Finally, the personal registration information of 1000 users is selected in the data set, and the experiment process is as follows:

  Step 1: import the data set, and process the data to remove stop words
  Step 2: word segmentation, vectorize the experimental data completed by word segmentation
  Step 3: vectorize the category labels
  Step 4: divide the training and test data sets
  Step 5: build an algorithm model for experiment

**Experiment 2 .**

The recommendation algorithm based on the learner model and the traditional collaborative filtering recommendation algorithm on the DataALL dataset comparative experiment.

The experiment uses the DataALL dataset to compare the accuracy, novelty, and *F*1 comprehensive indicators of the traditional collaborative filtering recommendation algorithm and the recommendation algorithm based on the learner model proposed in this chapter. The experimental data is randomly divided into a training set and a test set for experiments. For the convenience of calculation, Top-N is set to 10. The experimental process is as follows:

  Step 1: analyze the user's historical information to obtain the user's historical interest tag
  Step 2: according to the interest tag, find the corresponding set of the same user
  Step 3: according to the user's learning ability level similarity score and the user's learning feature similarity score, the learner model similarity score is obtained
  Step 4: get the user set from Step 1 to form the “user-course” rating table, and calculate the rating similarity
  Step 5: according to the final similarity scores between users, the final similar user set of target users is obtained
  Step 6: use the unrated items of the target user in the set of similar users of the target user to predict and output the recommended results

### 4.3. Analysis of Experimental Results

#### 4.3.1. Analysis of the Results of Experiment One

The accuracy of different algorithms on the data set in this paper is shown in Table 4.

According to the experimental results in Table 4, as the proportion of training data increases, the accuracy of various control methods has improved. Among them, the accuracy of CNN is low compared with other methods; the overall performance of GRU method is better than CNN; and when the proportion of training set is low, the accuracy of LSTM method is greatly improved compared with the previous two methods, but with with the gradual increase in the proportion of training data, the accuracy of the method in this paper has improved more obviously. It can be seen that the method in this paper has better algorithm performance for application scenarios with a large amount of data. When applied to the field of education resource management, it can alleviate the cold start problem.

#### 4.3.2. Analysis of the Results of Experiment Two

**Figure 6:**
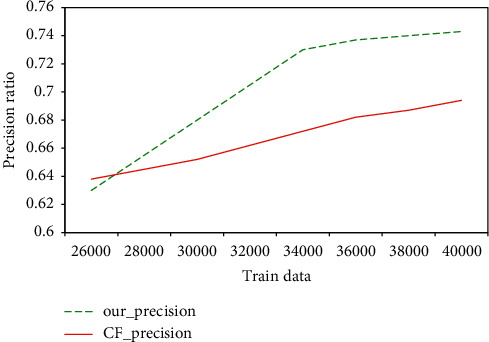
The accuracy of the learner model recommendation algorithm.

Figure 6 shows the accuracy results of the comparison experiment. The higher the accuracy, the better the resources recommended to the user in line with the user's interest. The dotted line in the figure represents the accuracy of the recommendation algorithm based on the learner model at various levels, and the solid line represents the accuracy of the traditional collaborative filtering algorithm at various levels. From the experimental results, it can be seen that in the case of relatively small training data the model in this article has too many conditions to filter out, and it is difficult to find users who have too many intersections with the target user, which leads to a low recommendation accuracy. However, as the data continues to increase, the accuracy of the algorithm in this article is on an upward trend. To a certain extent, the accuracy rate is better than the traditional collaborative filtering algorithm.

**Figure 7:**
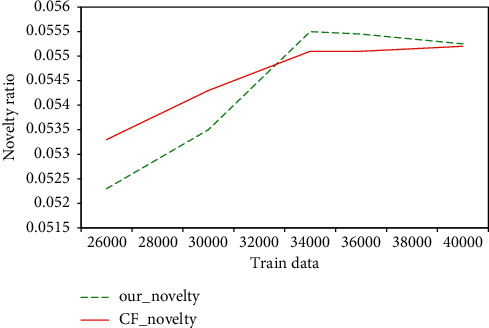
The novelty graph of the learner model recommendation algorithm.

Figure 7 is a graph of the novelty of the learner model recommendation algorithm. It can be seen from the graph that the method proposed in this paper is lower in novelty index than the traditional collaborative filtering algorithm in the case of a small number, but as the amount of data increases, the novelty of the recommendation method based on the learner model is gradually higher than that of the traditional collaborative filtering algorithm, which proves that the model in this paper has more advantages in recommending novelty than the traditional collaborative filtering in the big data scenario.

**Figure 8:**
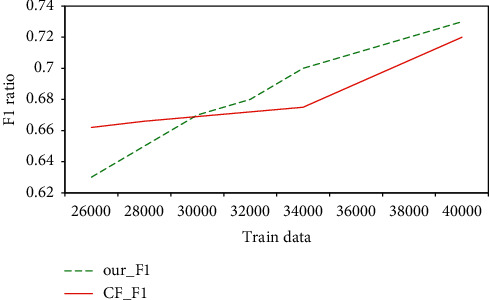
*F*1 comprehensive index diagram of learner model recommendation algorithm.

Since the accuracy rate only evaluates the learner model method from one aspect, in order to evaluate the comprehensive index of the algorithm based on the learner model, the *F*1 comprehensive evaluation index is used. It can be seen from Figure 8 that the *F*1 index is used in this article with less data. The proposed method is lower than the traditional collaborative filtering algorithm, but as the amount of data increases, the overall trend is increasing. The change curve of *F*1 of the recommendation method based on the learner model is gradually higher than that of the traditional collaborative filtering algorithm. It proves that the model in this paper is more suitable for scenarios with larger data.

Based on the above analysis, the recommendation method based on the learner model proposed in this paper has achieved significant results, alleviating the cold start problem of new users in the traditional recommendation system and introducing in-class calculations to improve the accuracy of resource recommendation for old users. At the same time, the model uses the objective characteristics of learners to calculate, which solves the problem of traditional collaborative filtering single score calculation.

## 5. Conclusion

This chapter mainly introduces the dynamic changes of learners' interest based on the MOOC platform. First, it introduces the introduction of dynamic changes in interest and the description and ideas of the recommendation algorithm; then it shows the overall structure of the algorithm and then briefly explains the structure; on this basis the implementation steps of the algorithm model are explained in detail. The algorithm mainly includes a combination of three situations. Tourist users directly perform popular resources and the latest resource fusion recommendation learning; new users use natural language processing to determine the category and then perform similarity calculations. Finally, calculate the scores of the resources that the target user may like based on the historical information of similar users and then recommend learning for them; the old users make recommendations by using the user's historical information. The difference is that this algorithm mainly focuses on the user's recent interests; thereby improving it can reflect the impact of short-term interest on the recommendation result, calculate the user's neighbor users through interest categories, learning capabilities, and resource ratings, and generate recommendation learning based on the target user's neighbor user information. Finally, the feasibility of the algorithm in this chapter is verified through design comparison experiments. It plays a more important role in the teaching management of the entire MOOC platform, but this algorithm also has certain limitations. Generally, it can only reflect short-term interests, and short-term interests will also affect the recommendation results.

## Figures and Tables

**Table 1:** The descriptions of the 11 learning behavior characteristics.

Label	Name	Illustration
D_1_	Number of courses visited	Number of visits to the course during the MOOC study
D_2_	Duration of the course	Duration of access to courses during MOOC study
D_3_	Access to other modules of the course	Number of visits to other modules of the course during the MOOC study
D_4_	Number of visits to the forum	Number of visits to assignments during MOOC learning
D_5_	Visits to the wiki	The number of visits to the forum corresponding to the course in the MOOC study course
D_6_	Total number of visits	Number of visits to Wikipedia during MOOC study
D_7_	Activities	Active days in the MOOC learning process
D_8_	Active days	The total number of learning activities in the MOOC learning process
D_9_	Page closed	The number of times the web page was closed during the MOOC learning process
D_10_	Video views	The number of times the instructional video was watched during the MOOC learning process
D_11_	Watch video time	The number of times the web page was closed during the MOOC learning process

**Table 2:** Learner characteristics.

	Personal information	Learning characteristics
New user	Registration message	Learning ability, learning time
		Learning experience, learning score

Old users	History study information	Study progress number of homework submissions
		Course score, learning ability

**Table 3:** User-rating table.

	*I*_1_	*I*_2_	*I*_3_	*I*_4_	*I*_5_
*U*_1_	2	5	7	1	3
*U*_2_	1	1	3	2	4
…	…	…	…	…	…

**Table 4:** Experimental results.

Test : train	CNN	GRU	LSTM	OURS
2 : 3	0.702	0.821	0.830	0.834
3 : 7	0.710	0.824	0.832	0.840
1 : 4	0.768	0.837	0.853	0.881

## Data Availability

The dataset can be accessed upon request.
